# Supplementary material for: Binder-type effect on the physico-mechanical, combustion and emission properties of Alstonia boonei De Wild. sawdust and Theobroma cacao L. pod biochar briquettes for energy applications
Source: PLoS One. 2024 Jul 29;19(7):e0306827. doi: 10.1371/journal.pone.0306827 (PMC11285954; doi:10.1371/journal.pone.0306827)
Supplement: Attachment — Submitted filename: Response to Reviewers.docx [file pone.0306827.s001.docx]

**Reviewer #1:**

Comments for the manuscript entitled “Binder-type effect on the physico-mechanical, combustion and emission properties of Alstonia boonei De Wild. sawdust and Theobroma cacao L. pod briquettes as bioenergy products” (MS ID: PONE-D-23-06748)

It is interesting to study the briquetization and characterization of biochar briquette produced from Alstonia boonei De Wild. sawdust and Theobroma cacao L. pod with different binders. However, some aspects are not clear. Thus, the comments for improving this manuscript are listed as below.

**Title**

- The authors should consider this modified title “Binder-type effect on the physico-mechanical, combustion and emission properties of Alstonia boonei De Wild. and Theobroma cacao L. pod biochar briquettes for energy applications”.

Authors’ response: This advice is taken and has been effected in the manuscript.

- The title should be clearly indicated as biomass briquettes or biochar briquettes.

Authors’ response: This advice is taken and the title has been re-written to capture ‘biochar briquettes’.

**Abstract**

- Try to avoid wording “Energy production and energy generation”. This is because energy can not be generated following the first law of Thermodynamics. If energy can be produced or generated, it will destroy that law. You may change as “energy application….”.

Authors’ response: This advice is taken and duly corrected.

- Type of combustor and combustion condition strongly affect the emissions both flue gas components and PM concentration, thus this information should be mentioned.

Authors’ response: These have been duly added in the abstract now. PAGE 2, LINE 38 & 39.

- Key results like energy properties should be reported in this section.

Authors’ response: Such have already been captured and updated. PAGE 2, LINE 40 - 49

**Introduction**

- The reason for why biochar briquette is more interesting than biomass briquette should be added in this section.

Authors’ response: These have been duly given now. PAGE 3, LINES 69 – 744; PAGE 4, LINE, 89; PAGE 5, LINE, 115 – 117.

- The order of reason or research motivation of this section should be revised. The logical order is required of this section.

Authors’ response: These have been considered and duly effected.

- The key properties of biochar briquette should be mentioned and which factors affect the biochar briquette properties. Many previous studies have done and reported about the biochar production and characterization.

Authors’ response: Such had been given briefly, and a little more added in this review. LINE PAGE 5, 119 - 125

- The reason (s) for why binder type strongly affects the biochar briquette properties both mechanical and energy aspects, as well as the criteria for selecting the biochar type.

Author's response: This has been clearly added now –LINE 121 – 123. The criteria for selecting the biochar type can be found from Line 84 – 100. They were selected based on the large volumes of the waste being produced.

**Materials and methods**

- The temperature for biochar production needs to be clearly indicated.

Author's response: This has been clearly added now – LINE 153-154.

- The authors should clearly indicate that this study uses biomass sawdust or biochar sawdust for briquetization.

Author's response: *T. cacao pod* husk and *A. boonei* sawdust were carbonized to produce biochar briquettes and this has been clearly stated at all points now.

- The determination of bulk density by water displacement is not suitable for biochar briquette. This is because this material absorbs water during dipping or soaking. Thus, the recorded volume or mass will be error. The non-wetting fluids are more suitable or the way of solid displacement by using very fine grass bead.

Author's response: This was considered during determination and rewritten to capture necessary steps taken to prevent moisture uptake. The biochar briquettes were tightly wrapped in light cling film (Everpack®) (negligible weight) to prevent water imbibition.

- The properties of raw materials should be determined and reported.

Author's response: The study sought to investigate the binder effect on the biochar properties, and not the biomass raw material briquette. With this in mind, the properties of the non-bindered samples (Controls) were determined and data reported.

- What is the basic density? Normally, the density of materials can be reported as bulk density or true density. It is the density of single briquette or briquettes, this requires the information.

Author's response: Basic density is different from bulk density, and averages of all data for the various properties are what is reported. Raw data from replications were used for data analysis.

- The authors did not determine the energy properties like gross components (proximate analysis), elemental components (ultimate analysis), heating value and energy density of the produced biochar briquette. These basic properties should be determined and reported.

Author's response: The authors had already determined energy properties like Specific fuel consumption, and burning rate. We have however now determined the Proximate properties and calorific values of the biochar briquettes and provided reports of them in the review.

- The burning test of biochar briquette performed at ambient air or suppling air. The condition should be clearly indicated because it strongly affects the burning rate.

Author's response: Ambient room temperature and air velocity of 25 ℃ and 0.8 m/s respectively – Line 284 & 285

- Do the authors dry the biochar briquette sample after briquetization before determining the properties? The moisture content of dried biochar briquette should be reported.

Author's response: The moisture content of the biochar briquettes has been reported.

- For biochar briquetization with binder, the temperature during the process is important for the adhesion of binder and biochar particle. This study did not mention about this issue.

Author's response: The clay and starch binders used do not need high or low temperatures to cure, both required just ambient temperature. Only wax paste was prepared by boiling and the temperature of 100℃ has now been indicated - LINE 165 - 167.

**Results and discussion**

- The authors need to check the unit of burning rate. The results showed that the value was in the range of 0.5-2.5 x 103 kg/min (Fig. 5), which is so high.

Author's response: The values have been checked and scale corrected.

- This study produced solid biofuel but the energy properties of the product are not reported. Thus, the authors should report them.

Author's response: Energy properties; calorific value, proximate properties, burning rate, and specific fuel consumption are reported.

- The authors reported the specific fuel consumption for boiling water. However, this point may not reply the useful energy of the product. This is because heat loss may be prominent during burning of biochar briquette.

Author's response: Their calorific value was reported and burning rate have been reported. The specific fuel consumption for boiling reported is very important not only for water heating but also in applications like cooking, barbecuing, and all such energy applications. The authors employed scientifically published and standard methods in all property determinations.

- The authors should give the reasons how binder type and biomass type affect the emission both CO and PM.

Author's response: This has been done.

- The key results should be clearly indicated and discussed.

Author's response: This has been done.

- Check the unit of density, for example, 490 kg/cm3 or it is 490 kg/m3?

Author's response: They are both S.I units and correct. The authors decided to go with the former.

**English**

- Carefully check the typing and format of typing. Some sentences have words with capital letter.

Author's response: All such have been done and corrected where necessary.

**Reviewer #2:**

The manuscript is well-prepared but significant improvements are necessary. Major revision is required. Authors can find the comments as follows:

1) Section 2.1: Carbonization temperature and heating rate should be reported.

Author's response: The carbonization temperature and heating rate (i.e., (at 410 ± 5°C, with a heating rate of 4°C/min from the ambient temperature of 25°C) has been reported now.

2) Citation is very excessive. In general, original research article should have not more than 50 references. Please recheck and remove some references that are not related to the text. For example, citation [43] has nothing to do with the basic density definition or method. Only reference [42] is enough. Also, reference [40] may not be necessary, instead the detail method of quenching and weighing can be described.

Author's response: This has done and the references reduced to 49.

3) It is better (if possible) to have real evidence by performing some analysis work instead of just citing the reference. For example, the structure or constituent of each binder should be analyzed so their characteristics that affect the property of the briquette can be investigated. For example, Page 11 Line 259-260, fatty acid molecule in wax gave the resulted briquette heavy and stable unlike the one made with starch. Also, authors discuss about pore size of the material. It is better to perform BET surface analysis of the samples instead of just citing the reference (Page 12, Line 281-282).

Author's response: The authors are of the view that evidential studies as suggested is apt, however, the references cited from scientific papers are also very much apt.

4) Figure 3 is strange. density values should not be presented with the water resistance capacity. They have different unit and meaning. Also, their values are significantly different; the scale should be adjusted appropriately. Figure 4 and 5 has the same issue.

Author's response: These figures have all now been represented separately.

5) Fracture toughness results are very interesting and unique. However, the discussion on the effect of pore size of clay is based on citation (Page 15, Line 336). It is better to have real pore size analysis and discuss with your own results. Also, Line 335 read "the lower the MC of the briquette, the greater is its fracture toughness [27]", is that your own results or it is only from the citation? Please clarify. If MC is the parameter during briquetting process, it should be mentioned in the methodology.

Author's response: The authors are of the view that evidential studies as suggested is apt, however, the references cited from scientific papers are also very much apt, thus adopted.

For the second part, it is from the citation.

For the third part, the MC of the biochar briquettes were determined and now reported accordingly.

6) What is a,b,c,d,e superscript in Table 2.

Author's response: They represent differences or not between the various treatments. “Bars/Means with the same letter(s) and numerals for each property are not significantly different (p>0.05).”

7) The conclusion should be rewritten. Some opening sentences are needed before bullet conclusions.

Author's response: This has been done.
