# Supplementary material for: Binder-type effect on the physico-mechanical, combustion and emission properties of Alstonia boonei De Wild. sawdust and Theobroma cacao L. pod biochar briquettes for energy applications
Source: PLoS One. 2024 Jul 29;19(7):e0306827. doi: 10.1371/journal.pone.0306827 (PMC11285954; doi:10.1371/journal.pone.0306827)
Supplement: Attachment — Submitted filename: Review plos one binder.docx [file pone.0306827.s002.docx]

This manuscript discussed char briquette made of two kinds of raw materials with different binder namely clay, starch, and wax. Some questions should be raised since the experiment on these products are less clear, for instances:

1. Why authors choose char-briquettes instead of briquette? This information should be included in introduction section.
2. Why authors use wax as the binder? It is not safe if the char briquettes are used as barbeques fuel!
3. It is better to include the chemical content of the raw materials (pod of cacao and sawdust of Alstonia wood) as well as after they become char.
4. The composition of mixture pod and sawdust was missing in the method.
5. The binder should be stated as weight/weight (w/w) or weight/volume (w/v).
6. The authors emphasized on binder but the fundamental characteristics of the binders (origin, chemical content, MSDS/ material safety data sheet, etc) were missing in the text.
7. Authors mentioned 10 replications for each type of the briquette. However, when I checked into the excel (supplementary materials), there were only three replications that authors did for testing.
8. Further, for statistical analysis, I could not find the complete analysis. They should be attached in the excel supplementary materials
9. Bio-char is related also as the absorbent instead of only for fuel. Why authors did not test for this purpose?
10. Please make the conclusion concise! Not point by point!
